# Hypomethylation of *FAM63B* in bipolar disorder patients

**DOI:** 10.1186/s13148-016-0221-6

**Published:** 2016-05-11

**Authors:** Anna Starnawska, Ditte Demontis, Andrew McQuillin, Niamh L. O’Brien, Nicklas H. Staunstrup, Ole Mors, Anders L. Nielsen, Anders D. Børglum, Mette Nyegaard

**Affiliations:** Department of Biomedicine, Aarhus University, Wilhelm Meyers Alle 4, DK- 8000 Aarhus C, Denmark; The Initiative for Integrative Psychiatric Research, iPSYCH, Aarhus, Denmark; Center for Integrative Sequencing, iSEQ, Aarhus University, Aarhus, Denmark; Molecular Psychiatry Laboratory, Division of Psychiatry, Rockefeller Building, University College London, London, UK; Psychosis Research Unit, Aarhus University Hospital, Risskov, Denmark; Translational Neuropsychiatry Unit, Aarhus University Hospital, Risskov, Denmark

**Keywords:** Epigenetics, DNA methylation, Candidate gene, Mental disorder, iPLEX, *FAM63B*, Bipolar disorder

## Abstract

Bipolar disorder (BD) and schizophrenia (SZ) are known to share common genetic and psychosocial risk factors. A recent epigenome-wide association study performed on blood samples from SZ patients found significant hypomethylation of *FAM63B* in exon 9. Here, we used iPLEX-based methylation analysis to investigate two CpG sites in *FAM63B* in blood samples from 459 BD cases and 268 controls. Both sites were significantly hypomethylated in BD cases (lowest *p* value = 3.94 × 10^−8^). The methylation levels at the two sites were correlated, and no strong correlation was found with nearby single nucleotide polymorphisms (SNPs), suggesting that methylation differences at these sites are not readably picked up by genome-wide association studies. Overall, *FAM63B* hypomethylation was found in BD patients, thus replicating the initial finding in SZ patients. This study suggests that *FAM63B* is a shared epigenetic risk gene for the two disorders.

**Electronic supplementary material:**

The online version of this article (doi:10.1186/s13148-016-0221-6) contains supplementary material, which is available to authorized users.

## Introduction

**Fig. 1:**
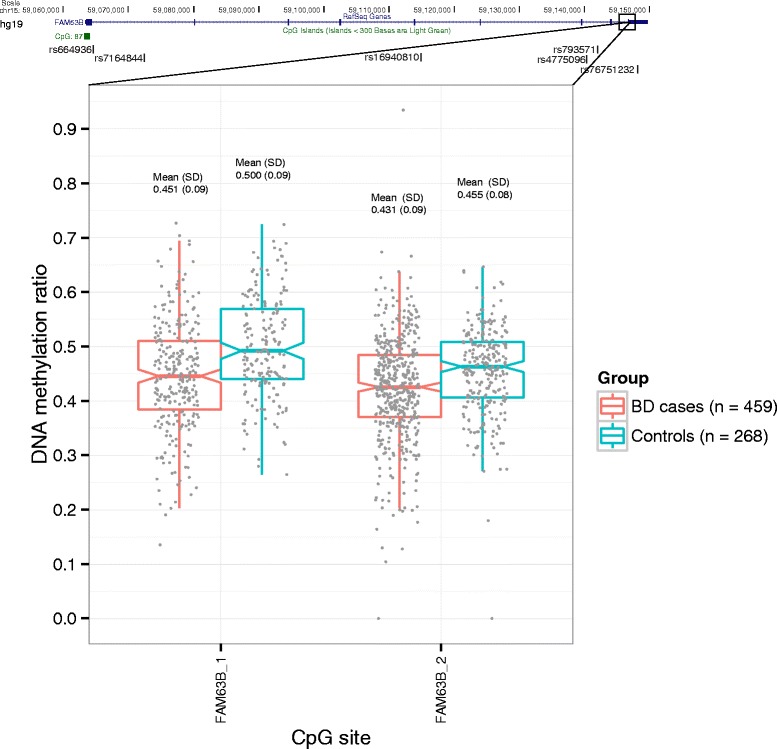
DNA methylation levels in cases and controls of two CpG sites in *FAM63B* exon 9 (*lower panel*). Gene structure of *FAM63B* and position of six relevant SNPs (four genotyped from Affymetrix 500K array, a significant *cis*-mQTL (rs76751232), and one SNP associated with SZ in a genome-wide association analysis (rs793571) [21]. The upper and lower hinges correspond to the 25th and 75th percentiles, while the whiskers extend from hinges to the highest and lowest values within 1.5 * IQR (inter-quartile range)

Bipolar disorder (BD) and schizophrenia (SZ) are severe complex mental disorders known to have common genetic [1] and psychosocial risk factors [2], as well as overlap in some of their symptoms [3]. Together they are classified as major psychosis and contribute significantly to the global burden of disease [4]. Several studies have investigated differences in DNA methylation between individuals with major psychosis and healthy controls in various tissues and associated this epigenetic modification with psychiatric phenotype [5–8]. A recent sequencing-based epigenome-wide association study (EWAS) performed on blood samples from 759 SZ cases and 738 control individuals identified differential DNA methylation in family with sequence similarity 63, member B (*FAM63B*) gene in a region of exon 9 as their top EWAS finding (*p* value = 6.3 × 10^−11^), thereby becoming the first to implicate this gene in a psychiatric disorder [9]. In the same publication, the authors then used targeted bisulfite pyrosequencing of three specific CpG sites in an 18 bp region of *FAM63B* to follow up the top hit in an independent replication sample of more than a thousands individuals. For all three CpG sites, they obtained independent replication with *p* values ranging from 2.76 × 10^−12^ (first CpG site in 18 bp region*)* to 2.31 × 10^−10^ (last site in the 18 bp region) [9]. The *FAM63B* gene spans 86 kb on chromosome 15q22.1, contains nine exons in total and one CpG island that overlaps with exon 1 (Fig. 1). The role of *FAM63B* in the pathophysiology of SZ is unclear. Due to the high degree of comorbidity between SZ and BD, as well as the established evidence for shared genetic risk factors for the two disorders [1, 10], it is highly relevant to investigate if the CpG sites in SZ risk locus in *FAM63B* demonstrates the same methylation pattern in BD. The aim of the study was to determine if methylation *FAM63B* is associated with BD. We used the Sequenom MassARRAY assays targeting specifically the two CpG sites identified as the sites of most interest in SZ [9] to analyze DNA derived from whole blood from 459 BD patients and 268 control individuals.

## Methods

### Study population

Bipolar disorder patients and control individuals were collected at UCL and collaborating clinical centers. Cases (*n* = 459) were Caucasian individuals who received clinical diagnoses of BD according to UK National Health Service (NHS) psychiatrists at interview using the categories of the International Classification of Disease version 10 (ICD10). Control individuals (*n* = 268) were recruited from London branches of the National Blood Service, from local NHS family doctor clinics, and from university student volunteers. All control individuals were interviewed with the Schizophrenia and Affective Disorders Schedule-Life Time version to exclude all psychiatric disorders [11]. The sample is partly overlapping with the UCL sample described in Sklar et al. [12]. The overlap is defined as all individuals from the UCL sample where DNA was still available and where the DNA was extracted from whole blood (*n* = 587). Additional cases and controls were included using similar inclusion criteria (*n* = 140), reaching a sample size of 727 participants. For a description of the sample see Additional file 1: Table S1.

### Ethics, consent, and permissions

The project was approved by the Metropolitan Multicenter Research Ethics Committee, and all participants provided written informed consent.

### Methylation methods

Genomic DNA was bisulfite converted with the use of EZ DNA Methylation Kit (Zymo Research, Freiburg, Germany). The MassARRAY Designer tool in Typer 4.0 (Sequenom, San Diego, CA, USA) was used to design independent iPLEX assays for the same CpG sites as investigated in the Swedish EWAS study [9]. The FAM63B_1 assay targeted CpG at position chr15:5,9146,738 (hg19) (corresponding to the *first site in 18 bp region* in Aberg et al.), and FAM63B_2 targeted CpG site at position chr15:59,146,756 (hg19) (corresponding to the *last site in 18 bp region* in Aberg et al. [9]). The two CpG sites are positioned 18 bp apart in exon 9 of *FAM63B* and are not part of a CpG island (Fig. 1). Primers for iPLEX assays can be found in Additional file 2: Table S2. Bisulfite converted DNA samples were prepared according to the iPLEX manufacturer’s protocol. The converted DNA was dispensed onto a SpectroCHIPII and analyzed on a MassARRAY workstation in the Allelotype mode (Sequenom). Eighteen samples were independently bisulfite converted and analyzed in duplicates for the FAM63B_2 assay. This analysis demonstrated a good overall consistency between duplicate samples (Additional file 3: Figure S1). Additionally six samples were subjected to pyrosequencing, demonstrating that the variation in methylation levels for FAM63B_2 between individuals was also evident with a different technology (Additional file 3: Figure S1). A regression analysis including BD group status and gender was initially performed on logit-transformed methylation. As the age distribution was different between cases (mean = 47.7, SD = 12.4) and controls (mean = 35.7, SD = 13.5), we tested for the effect of age using regression analysis on logit-transformed methylation in the control group and case group separately. In addition, a comparison of DNA methylation between cases and controls was performed using a nonparametric Mann-Whitney test. Each CpG site was analyzed independently, as well as in combination as a differentially methylated region (DMR) by taking the average of the methylation level of the two sites for each individual. Finally, patients with bipolar I and II disorders were compared using a nonparametric Mann-Whitney test.

### Genotypes, PCA, imputing, and mQTL analysis

Genotypes from the Affymetrix Gene Chip Human Mapping 500K Array were available for 587 of the individuals, as they were part of a published genome-wide association study [12]. Principle component analysis (PCA) was performed using SmartPCA [13] with an LD-pruned set of single nucleotide polymorphisms (SNPs) (*R*^2^ < 0.1, 39918 independent SNPs).

For *cis* methylation Quantitative Trait Loci (mQTL) analysis, SNPs in *FAM63B* and an additional 5 kb up- and downstream were imputed using Markov Chain Haplotyping software (MACH) [14]. Imputation was performed on data from four genotyped SNPs in *FAM63B* (rs664936, rs7164844, rs16940810 and rs4775096, all indicated at the top of Fig. 1) and the 1000Genome phase 1 reference panel. Genotype counts for the four genotyped SNPs can be found in Additional file 4: Table S3. Imputed SNPs were filtered by removing monomorphic markers and those with an RSQ score ≤0.3. This resulted in a final dataset of 173 imputed SNPs. To test for *trans*-mQTL we performed an additional analysis that included 9071 genotyped SNPs on chromosome 15. For the *trans*-mQTL analysis we filtered the results using an unadjusted *p* value <0.05 and Δβ ≥ 0.1. All mQTL analyses were performed using linear regression in PLINK [15].

## Results

A regression analysis of methylation levels found significant effect of affection status (*p* value = 7.92 × 10^−8^ for FAM63B_1 and *p* value = 3.06°×°10^−3^ for FAM63B_2), and no effect of gender (*p* value = 0.41 for FAM63B_1 and *p* value = 0.57 for FAM63B_2). No significant effect of age on methylation was found in a regression analysis among the controls (lowest *p* value = 0.48) or cases (lowest *p* value = 0.30).

In a direct comparison of cases and controls with Mann-Whitney test we found significant DNA hypomethylation in BD cases compared to controls (FAM63B_1 *p* value = 3.94 × 10^−8^, Δβ = −0.05 and FAM63B_2 *p* value = 8.59 × 10^−7^, Δβ = −0.02) (Fig. 1). Consistent with these CpG sites being located 18 bp apart, the methylation levels at the two sites were significantly correlated (Spearman correlation rho = 0.64, *p* value <2.2 × 10^−16^). When analyzing the two sites combined as a DMR, the region also displayed significant hypomethylation in BD (*p* value = 6.85 × 10^−6^, Δβ = −0.04). Overall, our BD data replicates the *FAM63B* hypomethylation findings that had been reported in SZ.

The PCA found no evidence of population stratification among the genotyped individuals (Additional file 5: Figure S2), suggesting the differences were not driven by ancestry stratification. No methylation differences were found between bipolar I and bipolar II disorder patients, but because of the small number of individuals with a bipolar II diagnose, this may not be generalizable.

To investigate whether methylation levels were correlated with nearby genotypes, we carried out mQTL analysis. In the *cis*-mQTL analysis, using only SNPs in the near neighborhood, we found no significant mQTLs with strong effects (β threshold ≥0.1). The most significant mQTL was found in exon 9 (imputed SNP rs76751232) for *FAM63B_2* (unadjusted *p* value = 0.011 and Δβ = −0.025 for the G allele). The full *cis*-mQTL analysis can be found in Additional file 6: Table S4. In order to assess the effect of *trans*-mQTLs, which are less frequent but can be notable [16], an mQTL analysis was performed including 9071 genotyped SNPs on chromosome 15. Again using the thresholds (*p* value <0.05 and Δβ ≥ 0.1) analysis revealed one nominally significant mQTL for FAM63B_1 (rs4774353 in *FOXB1*, chr15:60309674 (hg19), with *p* value = 0.006 and Δβ = −0.11 for the A allele). Overall, this suggests that the *FAM63B* methylation differences seen in whole blood from BD and controls are not strongly driven by SNPs in *FAM63B*, at least those that are on the Affymetrix 500K array. Of note, a potential mQTL may exist a few genes upstream, in *FOXB1*, which needs further validation, as it can also be a false positive resulting from multiple testing.

Due to the relative large variation in methylation levels observed in both groups, confirmed by technical replicates and pyrosequencing, we speculated if differences in blood cell composition could influence *FAM63B* methylation. In the absence of methylation array data to estimate cell composition in our sample, we instead downloaded a publicly available Illumina 450K array methylation dataset for nine different cell types from human whole blood [17]. The nearest probe cg21149266 (chr15:59,146,882 (hg19)) was positioned 126 bp downstream (3’) to FAM63B_2. Although not ideal, the methylation level of cg21149266 may for this purpose serve as a reasonable proxy for our two CpG sites, as methylation status of closely positioned sites is often correlated. In the cell-specific dataset, the methylation levels for cg21149266 were found to vary markedly between cell types with highest level in B cells, T cells, and NK cells, and lowest in monocytes, granulocytes and neutrophils (Additional file 7: Figure S3). It is thus possible that variation in cell composition contributes to the variation in *FAM63B* methylation observed in the entire dataset. If cell type heterogeneity is a systematic confounder between cases and controls, the effect should be shared across BD and SZ.

## Discussion

We report *FAM63B* methylation to be significantly associated with BD, thus replicating the same finding recently published for SZ [9], with consistency in genomic position (same CpG sites) and direction (lower methylation in cases) across both studies. This is of interest in the light of phenotypic overlap between BD and SZ [18], with relatives to BD patients having increased risk for developing SZ, schizoaffective disorder or major depression [19, 20]. It has also been shown that the two disorders share common genetic risk variants, with a SNP co-heritability estimate of 0.15 [1]. The discovery of shared epigenetic risk loci across the two diseases, with differential methylation at the same sites and in the same direction in the same tissue, as in the case of *FAM63B*, may help progress the understanding of the shared etiology.

Interestingly, a large recent genome-wide association study (GWAS) meta-analysis of SZ identified association with *FAM63B* SNP, rs793571, as the most associated finding in this gene (*p* value = 4.54 × 10^−6^) [21]. Rs793571 is positioned in intron 7 of *FAM63B* (~5 kb from exon 9) and could potentially affect methylation levels of the 18 bp region of interest. In our data, rs793571 was however not an mQTL for the two CpG sites and thus unlikely to be involved in the methylation differences in the 18 bp region of interest in blood, at least in our sample.

In general, we do not find strong local mQTLs for the two investigated CpG sites in blood. In human fetal brain, a recent study characterized systematically mQTLs and found no Bonferroni-corrected mQTLs for *FAM63B* [16]; however, larger samples sizes across different tissues may reveal robust mQTLs for *FAM63B*. The present literature and data does in our opinion not imply that methylation of this gene is strongly driven by genotypes of known common SNPs, suggesting that methylation differences at these sites are not readably picked up by GWAS. In the near future, dense genotyping coupled with DNA methylation and expression levels across different tissue and cell types from projects like PsychENCODE [22] will help to resolve whether the differences are driven by genetic variation or if they represent independent epigenetic signal.

The strength of the study was the use of the same tissue and analysis of the exact same CpG sites, as identified as the top differentially methylated sites in the large EWAS of SZ [9]. Also, we used a relatively large sample of bipolar cases and controls, with genotypes available for the majority of participants.

A limitation of this study is the possible confounder of age. Because the BD cases in our study were slightly older than controls, we analyzed the controls and cases separately and found no effect of age on methylation at this locus. Also, because the *FAM63B* hypomethylation was initially identified in a large EWAS study of SZ [9], and replicated in an independent sample, while adjusting for age, we find it unlikely that the hypomethylation in our study is driven by this covariate.

A second limitation is the possible confounder of blood cell composition on methylation that we were not able to correct for because of the candidate-gene approach in this study. This limitation is not specific to our study. While we acknowledge that cell heterogeneity may explain some of the variation in the entire dataset (also among controls), it becomes a confounder only if cell composition is systematically different in cases. If cell composition is a confounder, it must be shared between BD and SZ.

Thirdly, medication could influence the methylation levels of *FAM63B*. Interestingly, the use of mood-stabilizing medication has been shown to influence the DNA methylation patterns in blood in a recent study by Houtepen and co-workers [23], using primarily the Infinium HumanMethylation27 to measure methylation levels. This array does not cover sites in *FAM63B*, so the effect of medication on *FAM63B* methylation in BD (and SZ) remains to be determined. It would be of great interest in the future to collect medication information (drug type, dosage and patient response). This not only would permit correction for possible medication effects in methylation data but also would also allow separation of the BD patients into pharmacological subphenotypes that could provide a link between methylation signatures and medication response.

The biological function of *FAM63B* is currently not well described. The gene is expressed across most tissues, with highest expression in the cerebellar hemisphere and cerebellum according to the genotype-tissue expression (GTEx) portal [24] (Additional file 8: Figure S4). In the BioGPS reference panel of normal human tissues [25], high *FAM63B* expression was specifically apparent in the pineal body (Additional file 9: Figure S5), a small endocrine gland positioned in the center of the brain. Interestingly, the pineal body is known to be involved in producing and releasing melatonin which is involved in the circadian clock [26]. Disruption of the circadian clock has been linked to the etiology of multiple psychiatric disorders [27]. In the future, inclusion of information on diurnal mood variation and sleeping patterns of investigated individuals could allow for investigation of possible correlations between DNA methylation levels of *FAM63B* and the circadian clock.

In conclusion, we have identified *FAM63B* hypomethylation in BD. Our data supports that *FAM63B* is a shared epigenetic risk gene for BD and SZ.

## Additional files

Additional file 1: Table S1. Overview of participants in the study. (DOCX 42 kb) Additional file 2: Table S2. Primer sequences for the two *FAM63B* iPLEX assays. (DOCX 43 kb) Additional file 3: Figure S1. Technical assessment of the iPLEX method. Samples analyzed with iPLEX and pyrosequencing for FAM63B_2 site (panel A). Technical replicates for the FAM63B_2 assay run with iPLEX (panel B). (PDF 150 kb) Additional file 4: Table S3. Overview for genotype counts for SNPs genotyped in *FAM63B*. (DOCX 39 kb) Additional file 5: Figure S2. Scatterplot of PC1 and PC2 obtained from PCA of genome-wide independent SNPs (*n* = 39918, *R* ^2^ < 0.1) in UCL sample. (EPS 801 kb) Additional file 6: Table S4. Overview of all genotyped and imputed SNPs in *FAM63B* (plus 5 kb up- and downstream region) used in the *cis*-mQTL analysis of the two investigated *FAM63B* CpG sites. (DOCX 178 kb) Additional file 7: Figure S3. DNA methylation levels for cg21149266, an Illumina 450 K methylation array probe in the region of interest in *FAM63B* across different blood cell types [17]. (PNG 242 kb) Additional file 8: Figure S4. Expression of *FAM63B* across different tissues according to the GTEx portal (http://www.gtexportal.org/home/). (PDF 185 kb) Additional file 9: Figure S5. Expression of *FAM63B* across tissues according to BioGPS portal (http://biogps.org/). (PNG 137 kb)

## Abbreviations

BD: bipolar disorder
DMR: differentially methylated region
DNA: deoxyribonucleic acid
EWAS: epigenome-wide association study
FAM63B: family with sequence similarity 63, member B
GTEx: genotype-tissue expression (GTEx)
GWAS: genome-wide association study
MACH: Markov Chain Haplotyping software
mQTL: methylation quantitative trait loci
PCA: principal component analysis
SNP: single nucleotide polymorphism
SZ: schizophrenia
UCL: University College London
