# Supplementary material for: Hypomethylation of FAM63B in bipolar disorder patients
Source: Clin Epigenetics. 2016 May 11;8:52. doi: 10.1186/s13148-016-0221-6 (PMC4865008; doi:10.1186/s13148-016-0221-6)
Supplement: Additional file 1: Table S1. — Overview of participants in the study. (DOCX 42 kb) [file 13148_2016_221_MOESM1_ESM.docx]

**Table S1**. Overview of participants in the study

| **Samples** | **BD cases** | **Controls** | **Number of individuals in total** |
| --- | --- | --- | --- |
| **Samples with methylation** | 459 | 268 | 727 |
| **Samples with Affymetrix 500K Genotypes** | 357 | 230 | 587 |
| **Males/Females** | 184/275 | 132/136 | 727 |
| **Bipolar I/Bipolar II** | 426/25 | - | 451 |
| **Mean age in years (SD)** | 47.7 (12.4) | 35.7 (13.5) |  |
