# Supplementary material for: Hypomethylation of FAM63B in bipolar disorder patients
Source: Clin Epigenetics. 2016 May 11;8:52. doi: 10.1186/s13148-016-0221-6 (PMC4865008; doi:10.1186/s13148-016-0221-6)
Supplement: Additional file 2: Table S2. — Primer sequences for the two FAM63B iPLEX assays. (DOCX 43 kb) [file 13148_2016_221_MOESM2_ESM.docx]

**Table S2**. Primer sequences for the two *FAM63B* iPLEX assays

| **Primer** | **Primer sequence (5’ – 3’)** |
| --- | --- |
| FAM63B_1_F | ACGTTGGATGAAGTGGAAGATAATTTGGG |
| FAM63B_1_R | ACGTTGGATGTTCCAACAAAAACCAACAC |
| FAM63B_1_EXT | GAAGATAATTTGGGAATAGTGAA |
| FAM63B_2_F | ACGTTGGATGTTCCAACAAAAACCAACAC |
| FAM63B_2_R | ACGTTGGATGGGAAGATAATTTGGGAATAG |
| FAM63B_2_EXT | TTTTCTTTTTCTTTATCTTTTTCTC |
