# Supplementary figures and images for: Hypomethylation of FAM63B in bipolar disorder patients
Source: Clin Epigenetics. 2016 May 11;8:52. doi: 10.1186/s13148-016-0221-6 (PMC4865008; doi:10.1186/s13148-016-0221-6)

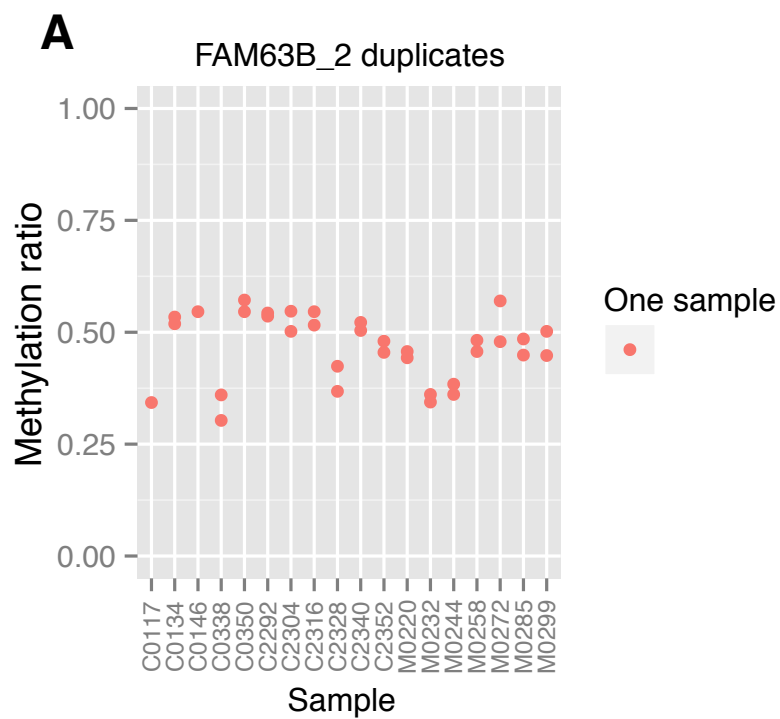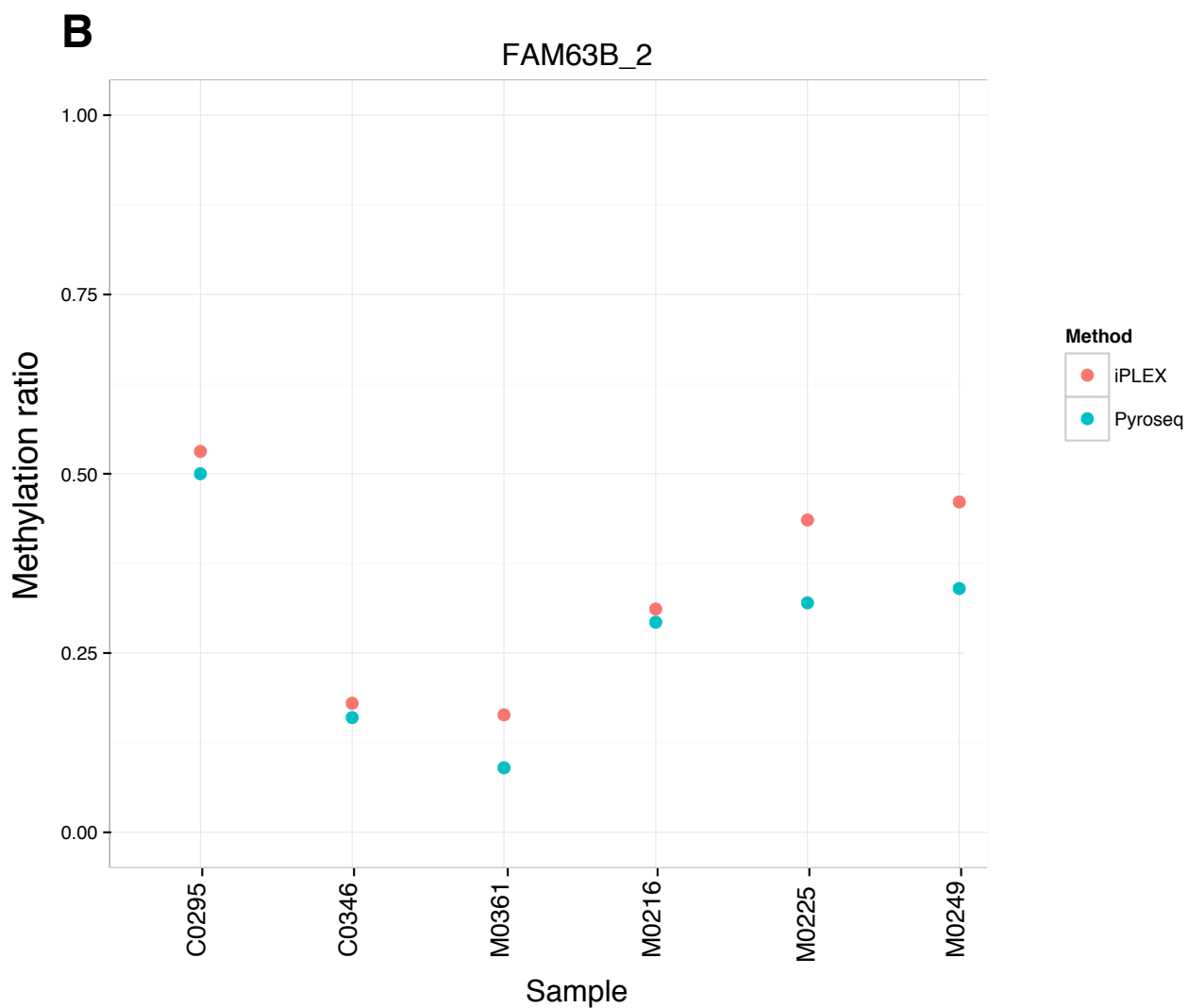

Supplement: Additional file 3: Figure S1. — Technical assessment of the iPLEX method. Samples analyzed with iPLEX and pyrosequencing for FAM63B_2 site (panel A). Technical replicates for the FAM63B_2 assay run with iPLEX (panel B). (PDF 150 kb) [file 13148_2016_221_MOESM3_ESM.pdf]

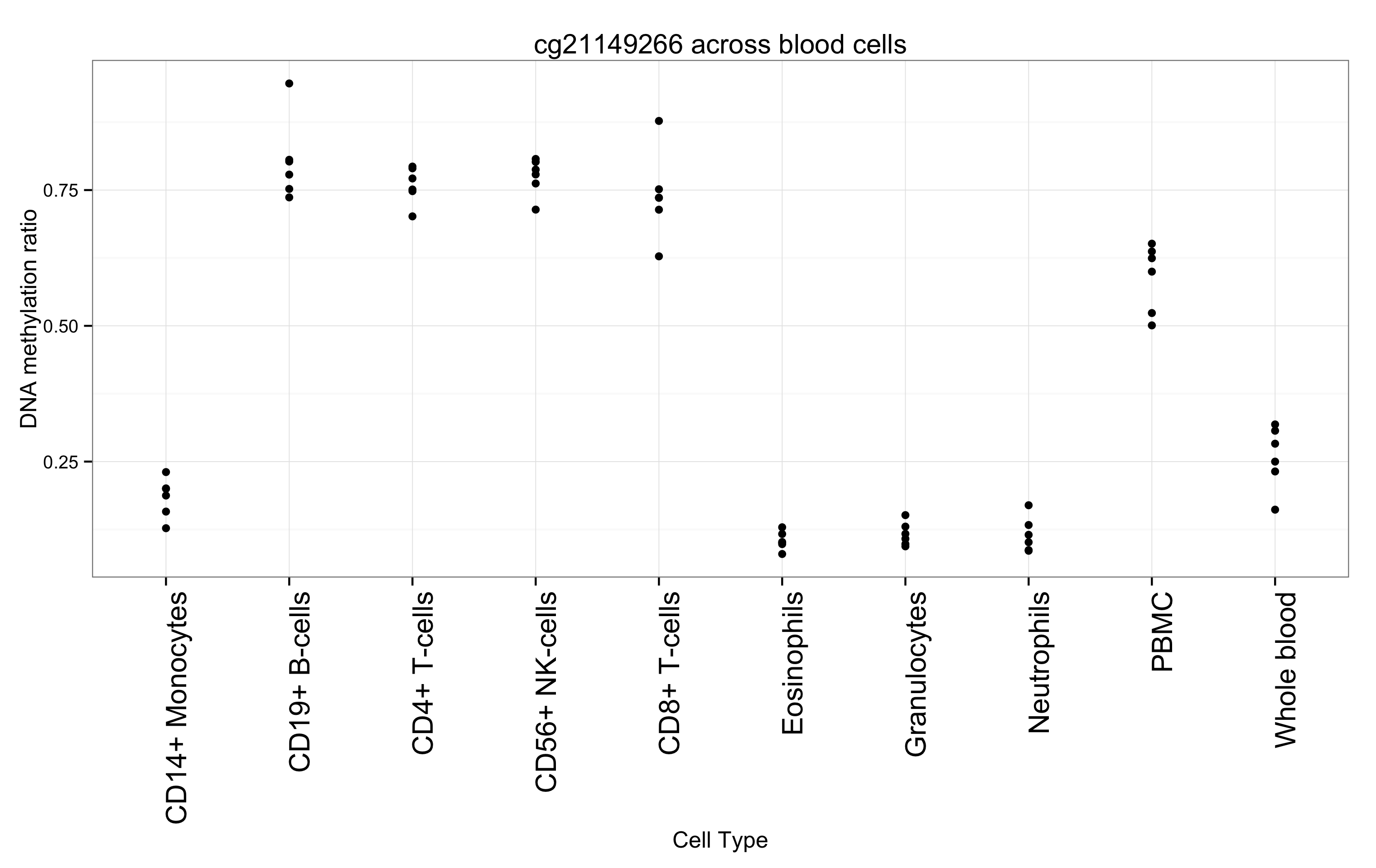

Supplement: Additional file 7: Figure S3. — DNA methylation levels for cg21149266, an Illumina 450 K methylation array probe in the region of interest in FAM63B across different blood cell types [17]. (PNG 242 kb) [file 13148_2016_221_MOESM7_ESM.png]

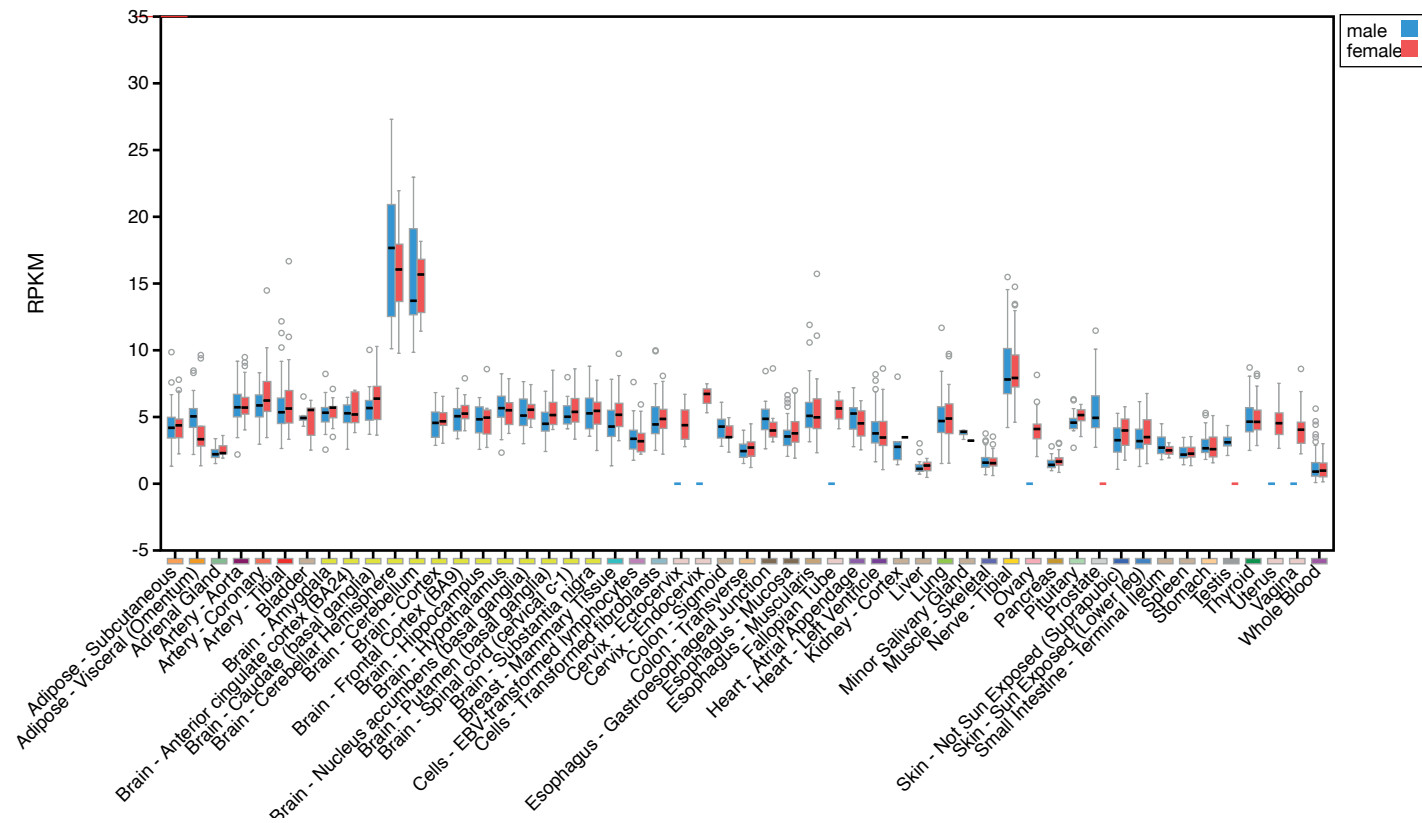

Supplement: Additional file 8: Figure S4. — Expression of FAM63B across different tissues according to the GTEx portal (http://www.gtexportal.org/home/). (PDF 185 kb) [file 13148_2016_221_MOESM8_ESM.pdf]

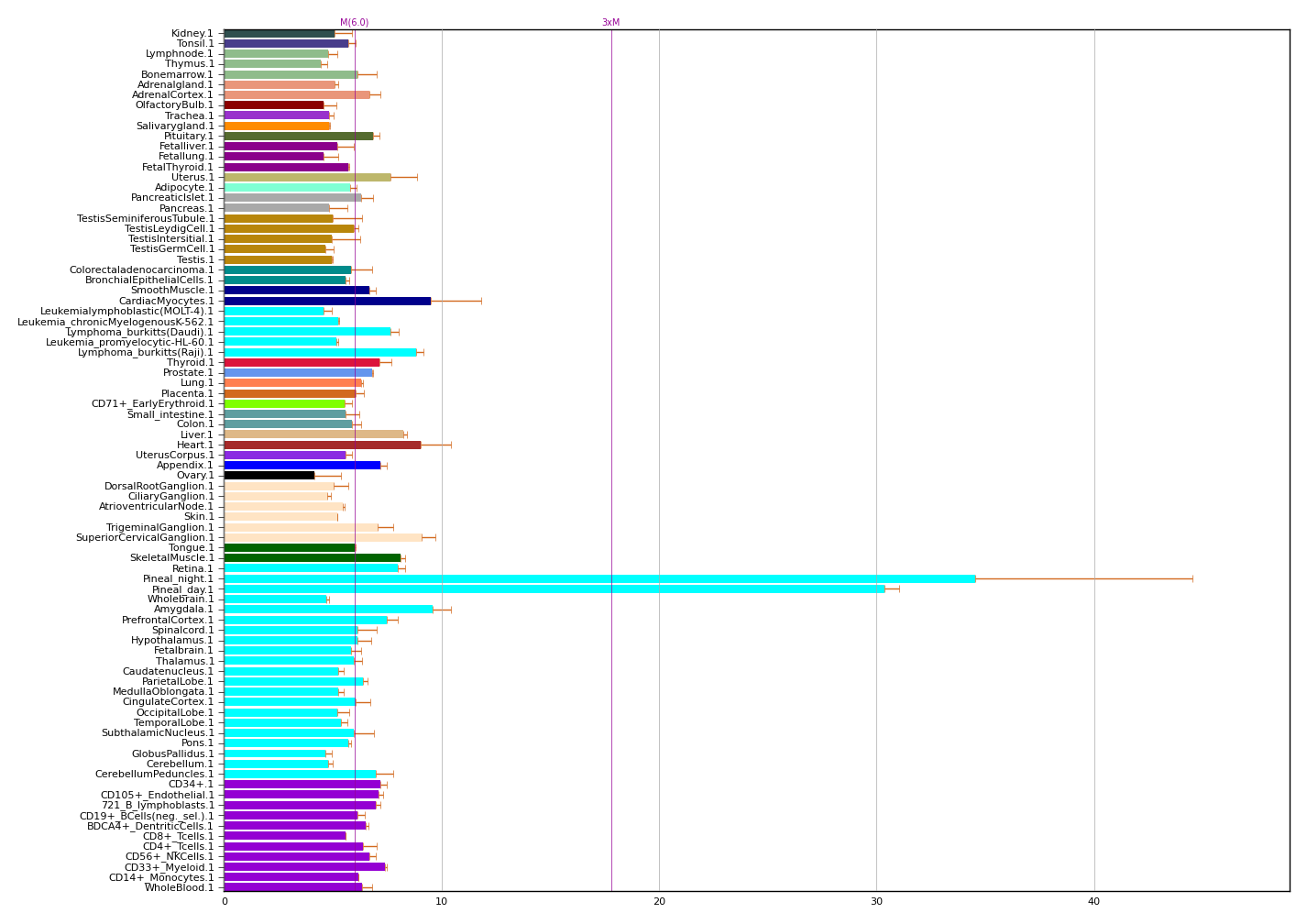

Supplement: Additional file 9: Figure S5. — Expression of FAM63B across tissues according to BioGPS portal (http://biogps.org/). (PNG 137 kb) [file 13148_2016_221_MOESM9_ESM.png]
