# Supplementary material for: Hypomethylation of FAM63B in bipolar disorder patients
Source: Clin Epigenetics. 2016 May 11;8:52. doi: 10.1186/s13148-016-0221-6 (PMC4865008; doi:10.1186/s13148-016-0221-6)
Supplement: Additional file 4: Table S3. — Overview for genotype counts for SNPs genotyped in FAM63B. (DOCX 39 kb) [file 13148_2016_221_MOESM4_ESM.docx]

**Table S3.** Overview for genotype counts for SNPs genotyped in *FAM63B*

| **SNP** | **A1** | **A2** | **BD cases (A1A1/A1A2/A2A2)** | **Controls**  **(A1A1/A1A2/A2A2)** |
| --- | --- | --- | --- | --- |
| **RS664936** | G | A | 14/124/217 | 11/67/149 |
| **RS7164844** | C | T | 9/96/251 | 5/55/170 |
| **RS16940810** | T | C | 0/15/341 | 0/12/217 |
| **RS4775096** | C | T | 27/139/188 | 25/102/99 |
