# Supplementary material for: Hypomethylation of FAM63B in bipolar disorder patients
Source: Clin Epigenetics. 2016 May 11;8:52. doi: 10.1186/s13148-016-0221-6 (PMC4865008; doi:10.1186/s13148-016-0221-6)
Supplement: Additional file 6: Table S4. — Overview of all genotyped and imputed SNPs in FAM63B (plus 5 kb up- and downstream region) used in the cis-mQTL analysis of the two investigated FAM63B CpG sites. (DOCX 178 kb) [file 13148_2016_221_MOESM6_ESM.docx]

**Table S4.** Overview of all genotyped and imputed SNPs in *FAM63B* (plus 5 kb up- and downstream region) used in the cis-mQTL analysis of the two investigated *FAM63B* CpG sites.

| **SNP** | **CHR** | **BP (hg19)** | **A1** | **Beta FAM63B_1** | **SE FAM63B_1** | **P-value FAM63B_1** | **Beta FAM63B_2** | **SE FAM63B_2** | **P-value FAM63B_2** | **SNP type** |
| --- | --- | --- | --- | --- | --- | --- | --- | --- | --- | --- |
| **RS347121** | 15 | 59059056 | G | -0.025 | 0.023 | 0.291 | 0.007 | 0.018 | 0.692 | Imputed |
| **RS650366** | 15 | 59061142 | G | -0.01 | 0.007 | 0.171 | -0.01 | 0.005 | 0.062 | Imputed |
| **RS11858548** | 15 | 59061724 | C | 0.007 | 0.018 | 0.711 | -0.015 | 0.012 | 0.21 | Imputed |
| **RS28533385** | 15 | 59062081 | A | 0.012 | 0.009 | 0.196 | -0.006 | 0.007 | 0.32 | Imputed |
| **RS16940766** | 15 | 59063137 | T | 0.017 | 0.097 | 0.862 | 0.002 | 0.086 | 0.978 | Imputed |
| **RS17269439** | 15 | 59063144 | A | -0.012 | 0.043 | 0.79 | 0.017 | 0.031 | 0.574 | Imputed |
| **RS395601** | 15 | 59063196 | C | -0.004 | 0.007 | 0.524 | -0.004 | 0.005 | 0.399 | Imputed |
| **RS16940768** | 15 | 59063506 | A | 0.017 | 0.097 | 0.862 | 0.002 | 0.086 | 0.978 | Imputed |
| **RS8029869** | 15 | 59064278 | A | 0.017 | 0.097 | 0.862 | 0.002 | 0.086 | 0.978 | Imputed |
| **RS664936** | 15 | 59064786 | G | 0.007 | 0.009 | 0.404 | -0.008 | 0.006 | 0.205 | Genotyped |
| **RS17301982** | 15 | 59064965 | T | 0 | 0.013 | 0.991 | -0.018 | 0.009 | 0.038 | Imputed |
| **RS652193** | 15 | 59065314 | A | 0.007 | 0.009 | 0.404 | -0.008 | 0.006 | 0.205 | Imputed |
| **RS11639208** | 15 | 59065624 | A | -0.025 | 0.023 | 0.291 | 0.007 | 0.018 | 0.692 | Imputed |
| **RS650731** | 15 | 59065659 | C | 0.012 | 0.015 | 0.394 | 0.019 | 0.011 | 0.101 | Imputed |
| **RS16940771** | 15 | 59066335 | G | 0.017 | 0.097 | 0.862 | 0.002 | 0.086 | 0.978 | Imputed |
| **RS11630503** | 15 | 59066539 | T | 0.007 | 0.018 | 0.711 | -0.015 | 0.012 | 0.21 | Imputed |
| **RS8025383** | 15 | 59066676 | T | -0.014 | 0.01 | 0.166 | 0.001 | 0.007 | 0.939 | Imputed |
| **RS7170538** | 15 | 59067687 | A | 0.004 | 0.011 | 0.705 | -0.018 | 0.007 | 0.013 | Imputed |
| **RS17236243** | 15 | 59067804 | A | 0.004 | 0.011 | 0.705 | -0.018 | 0.007 | 0.013 | Imputed |
| **RS7171198** | 15 | 59067860 | C | 0.004 | 0.011 | 0.705 | -0.018 | 0.007 | 0.013 | Imputed |
| **RS58937576** | 15 | 59068574 | G | 0.017 | 0.097 | 0.862 | 0.002 | 0.086 | 0.978 | Imputed |
| **RS17190720** | 15 | 59069370 | G | -0.025 | 0.023 | 0.291 | 0.007 | 0.018 | 0.692 | Imputed |
| **RS406699** | 15 | 59069854 | A | -0.001 | 0.01 | 0.934 | -0.015 | 0.007 | 0.029 | Imputed |
| **RS9646204** | 15 | 59070429 | A | 0.004 | 0.011 | 0.705 | -0.018 | 0.007 | 0.013 | Imputed |
| **RS7178970** | 15 | 59070918 | T | 0.004 | 0.011 | 0.705 | -0.018 | 0.007 | 0.013 | Imputed |
| **RS2019744** | 15 | 59071014 | C | 0.012 | 0.017 | 0.473 | 0.018 | 0.011 | 0.113 | Imputed |
| **RS379879** | 15 | 59071176 | G | -0.005 | 0.007 | 0.496 | -0.005 | 0.005 | 0.336 | Imputed |
| **RS4774311** | 15 | 59071581 | T | 0.004 | 0.011 | 0.705 | -0.018 | 0.007 | 0.013 | Imputed |
| **RS28733370** | 15 | 59071584 | G | -0.014 | 0.01 | 0.166 | 0.001 | 0.007 | 0.939 | Imputed |
| **RS7164844** | 15 | 59072201 | C | 0.004 | 0.011 | 0.705 | -0.018 | 0.007 | 0.013 | Genotyped |
| **RS7165019** | 15 | 59072297 | C | -0.014 | 0.01 | 0.166 | 0.001 | 0.007 | 0.939 | Imputed |
| **RS7164053** | 15 | 59072379 | A | 0.004 | 0.011 | 0.705 | -0.018 | 0.007 | 0.013 | Imputed |
| **RS7169796** | 15 | 59072491 | C | -0.014 | 0.01 | 0.166 | 0.001 | 0.007 | 0.939 | Imputed |
| **RS7168825** | 15 | 59072528 | C | -0.014 | 0.01 | 0.166 | 0.001 | 0.007 | 0.939 | Imputed |
| **RS625312** | 15 | 59073279 | G | 0.007 | 0.009 | 0.404 | -0.008 | 0.006 | 0.205 | Imputed |
| **RS17190727** | 15 | 59073790 | A | -0.008 | 0.007 | 0.283 | -0.009 | 0.005 | 0.102 | Imputed |
| **RS510759** | 15 | 59074536 | A | 0.007 | 0.009 | 0.404 | -0.008 | 0.006 | 0.205 | Imputed |
| **RS387812** | 15 | 59074786 | C | -0.004 | 0.007 | 0.524 | -0.004 | 0.005 | 0.398 | Imputed |
| **RS9744045** | 15 | 59074843 | C | 0.004 | 0.011 | 0.705 | -0.018 | 0.007 | 0.013 | Imputed |
| **RS596094** | 15 | 59075203 | C | 0.007 | 0.009 | 0.404 | -0.008 | 0.006 | 0.205 | Imputed |
| **RS561903** | 15 | 59075830 | G | 0.007 | 0.009 | 0.404 | -0.008 | 0.006 | 0.205 | Imputed |
| **RS11631334** | 15 | 59076200 | A | -0.023 | 0.023 | 0.316 | 0.006 | 0.017 | 0.713 | Imputed |
| **RS688756** | 15 | 59076272 | C | -0.005 | 0.007 | 0.496 | -0.005 | 0.005 | 0.336 | Imputed |
| **RS8032298** | 15 | 59076501 | T | 0.007 | 0.009 | 0.404 | -0.008 | 0.006 | 0.205 | Imputed |
| **RS8033478** | 15 | 59076611 | G | 0.004 | 0.011 | 0.705 | -0.018 | 0.007 | 0.013 | Imputed |
| **RS1648532** | 15 | 59076778 | C | -0.008 | 0.007 | 0.283 | -0.009 | 0.005 | 0.102 | Imputed |
| **RS673813** | 15 | 59077171 | T | -0.008 | 0.007 | 0.283 | -0.009 | 0.005 | 0.102 | Imputed |
| **RS411007** | 15 | 59078502 | C | -0.005 | 0.007 | 0.467 | -0.004 | 0.005 | 0.415 | Imputed |
| **RS421829** | 15 | 59079902 | C | -0.004 | 0.007 | 0.524 | -0.004 | 0.005 | 0.398 | Imputed |
| **RS7169205** | 15 | 59080203 | G | -0.014 | 0.01 | 0.166 | 0.001 | 0.007 | 0.939 | Imputed |
| **RS60162496** | 15 | 59080812 | C | 0.017 | 0.097 | 0.862 | 0.002 | 0.086 | 0.978 | Imputed |
| **RS384322** | 15 | 59080862 | A | -0.023 | 0.023 | 0.316 | 0.006 | 0.017 | 0.713 | Imputed |
| **RS28510910** | 15 | 59082387 | A | -0.014 | 0.01 | 0.166 | 0.001 | 0.007 | 0.939 | Imputed |
| **RS28617688** | 15 | 59082463 | C | 0.004 | 0.011 | 0.705 | -0.018 | 0.007 | 0.013 | Imputed |
| **RS347115** | 15 | 59082959 | T | -0.008 | 0.007 | 0.283 | -0.009 | 0.005 | 0.102 | Imputed |
| **RS28701980** | 15 | 59083374 | G | -0.014 | 0.01 | 0.166 | 0.001 | 0.007 | 0.939 | Imputed |
| **RS62002431** | 15 | 59083821 | G | -0.014 | 0.01 | 0.166 | 0.001 | 0.007 | 0.939 | Imputed |
| **RS12591310** | 15 | 59084268 | A | -0.014 | 0.01 | 0.166 | 0.001 | 0.007 | 0.939 | Imputed |
| **RS28470867** | 15 | 59084633 | T | -0.014 | 0.01 | 0.166 | 0.001 | 0.007 | 0.939 | Imputed |
| **RS28793354** | 15 | 59084846 | G | -0.014 | 0.01 | 0.166 | 0.001 | 0.007 | 0.939 | Imputed |
| **RS28800895** | 15 | 59085036 | C | -0.016 | 0.009 | 0.086 | 0.001 | 0.007 | 0.831 | Imputed |
| **RS34880782** | 15 | 59085522 | G | 0.007 | 0.009 | 0.404 | -0.008 | 0.006 | 0.205 | Imputed |
| **RS474875** | 15 | 59086703 | G | -0.008 | 0.007 | 0.283 | -0.009 | 0.005 | 0.102 | Imputed |
| **RS2555357** | 15 | 59087115 | G | -0.004 | 0.007 | 0.524 | -0.004 | 0.005 | 0.398 | Imputed |
| **RS12438114** | 15 | 59089464 | A | 0.007 | 0.009 | 0.404 | -0.008 | 0.006 | 0.205 | Imputed |
| **RS2604453** | 15 | 59089512 | C | -0.004 | 0.007 | 0.524 | -0.004 | 0.005 | 0.398 | Imputed |
| **RS601795** | 15 | 59090150 | G | 0.007 | 0.009 | 0.404 | -0.008 | 0.006 | 0.205 | Imputed |
| **RS34546804** | 15 | 59091003 | T | 0.004 | 0.011 | 0.705 | -0.018 | 0.007 | 0.013 | Imputed |
| **RS481309** | 15 | 59091965 | G | 0.007 | 0.009 | 0.404 | -0.008 | 0.006 | 0.205 | Imputed |
| **RS7183900** | 15 | 59092903 | T | 0.004 | 0.011 | 0.705 | -0.018 | 0.007 | 0.013 | Imputed |
| **RS12900320** | 15 | 59093110 | G | 0.007 | 0.009 | 0.404 | -0.008 | 0.006 | 0.205 | Imputed |
| **RS12901817** | 15 | 59093297 | C | 0.007 | 0.009 | 0.404 | -0.008 | 0.006 | 0.205 | Imputed |
| **RS72745027** | 15 | 59094329 | A | -0.005 | 0.007 | 0.468 | -0.006 | 0.005 | 0.283 | Imputed |
| **RS556256** | 15 | 59094764 | A | 0.007 | 0.009 | 0.404 | -0.008 | 0.006 | 0.205 | Imputed |
| **RS2266403** | 15 | 59096016 | G | 0.012 | 0.015 | 0.394 | 0.019 | 0.011 | 0.101 | Imputed |
| **RS2002386** | 15 | 59096033 | T | 0.01 | 0.017 | 0.551 | -0.014 | 0.012 | 0.231 | Imputed |
| **RS2013098** | 15 | 59096799 | C | -0.014 | 0.01 | 0.166 | 0.001 | 0.007 | 0.939 | Imputed |
| **RS28394222** | 15 | 59097157 | T | -0.013 | 0.01 | 0.176 | 0.001 | 0.007 | 0.916 | Imputed |
| **RS9788674** | 15 | 59097280 | A | 0.004 | 0.011 | 0.705 | -0.018 | 0.007 | 0.013 | Imputed |
| **RS512146** | 15 | 59099728 | T | 0.007 | 0.009 | 0.404 | -0.008 | 0.006 | 0.205 | Imputed |
| **RS16940795** | 15 | 59099949 | C | 0.017 | 0.097 | 0.862 | 0.002 | 0.086 | 0.978 | Imputed |
| **RS654870** | 15 | 59100344 | C | 0.007 | 0.009 | 0.404 | -0.008 | 0.006 | 0.205 | Imputed |
| **RS28812884** | 15 | 59100697 | G | 0.007 | 0.009 | 0.404 | -0.008 | 0.006 | 0.205 | Imputed |
| **RS2620360** | 15 | 59100837 | T | 0.007 | 0.009 | 0.404 | -0.008 | 0.006 | 0.205 | Imputed |
| **RS664783** | 15 | 59101104 | G | -0.021 | 0.022 | 0.348 | 0.006 | 0.017 | 0.713 | Imputed |
| **RS28412567** | 15 | 59102213 | A | -0.01 | 0.01 | 0.326 | 0.003 | 0.007 | 0.722 | Imputed |
| **RS28537903** | 15 | 59102279 | T | 0.004 | 0.011 | 0.705 | -0.018 | 0.007 | 0.013 | Imputed |
| **RS62002433** | 15 | 59102909 | G | -0.01 | 0.011 | 0.383 | 0.008 | 0.008 | 0.324 | Imputed |
| **RS610877** | 15 | 59103328 | A | -0.006 | 0.007 | 0.39 | -0.008 | 0.005 | 0.127 | Imputed |
| **RS71478692** | 15 | 59103460 | T | 0.026 | 0.097 | 0.786 | 0.147 | 0.086 | 0.089 | Imputed |
| **RS501165** | 15 | 59103483 | G | 0.007 | 0.009 | 0.404 | -0.008 | 0.006 | 0.205 | Imputed |
| **RS12439632** | 15 | 59103963 | C | -0.01 | 0.01 | 0.326 | 0.003 | 0.007 | 0.722 | Imputed |
| **RS36028781** | 15 | 59104413 | A | 0.026 | 0.097 | 0.786 | 0.147 | 0.086 | 0.089 | Imputed |
| **RS11634452** | 15 | 59104925 | G | 0.01 | 0.017 | 0.56 | -0.013 | 0.012 | 0.258 | Imputed |
| **RS452698** | 15 | 59105571 | G | -0.002 | 0.007 | 0.733 | -0.003 | 0.005 | 0.503 | Imputed |
| **RS1678977** | 15 | 59105655 | G | 0.007 | 0.009 | 0.404 | -0.008 | 0.006 | 0.205 | Imputed |
| **RS347112** | 15 | 59105778 | G | 0.007 | 0.009 | 0.401 | -0.008 | 0.006 | 0.208 | Imputed |
| **RS11631244** | 15 | 59106110 | G | -0.023 | 0.023 | 0.316 | 0.006 | 0.017 | 0.713 | Imputed |
| **RS7171482** | 15 | 59106495 | T | -0.01 | 0.01 | 0.326 | 0.002 | 0.007 | 0.726 | Imputed |
| **RS654857** | 15 | 59106744 | G | 0.007 | 0.009 | 0.41 | -0.008 | 0.006 | 0.207 | Imputed |
| **RS11071397** | 15 | 59107497 | C | -0.01 | 0.01 | 0.326 | 0.002 | 0.007 | 0.737 | Imputed |
| **RS12915713** | 15 | 59107862 | A | -0.012 | 0.009 | 0.182 | 0.003 | 0.007 | 0.645 | Imputed |
| **RS574683** | 15 | 59108316 | C | 0.007 | 0.009 | 0.41 | -0.008 | 0.006 | 0.207 | Imputed |
| **RS28889637** | 15 | 59109779 | A | -0.01 | 0.01 | 0.326 | 0.002 | 0.007 | 0.737 | Imputed |
| **RS597112** | 15 | 59110685 | G | 0.007 | 0.009 | 0.41 | -0.008 | 0.006 | 0.207 | Imputed |
| **RS28876251** | 15 | 59111320 | T | -0.01 | 0.01 | 0.326 | 0.002 | 0.007 | 0.737 | Imputed |
| **RS11629739** | 15 | 59111579 | T | 0.01 | 0.017 | 0.56 | -0.012 | 0.012 | 0.304 | Imputed |
| **RS688343** | 15 | 59112066 | T | 0.007 | 0.009 | 0.41 | -0.008 | 0.006 | 0.207 | Imputed |
| **RS34362744** | 15 | 59112470 | G | 0.007 | 0.009 | 0.41 | -0.008 | 0.006 | 0.207 | Imputed |
| **RS446126** | 15 | 59113102 | G | -0.002 | 0.007 | 0.732 | -0.004 | 0.005 | 0.463 | Imputed |
| **RS684505** | 15 | 59113642 | G | 0.007 | 0.009 | 0.41 | -0.008 | 0.006 | 0.207 | Imputed |
| **RS500057** | 15 | 59114475 | C | 0.007 | 0.009 | 0.41 | -0.008 | 0.006 | 0.207 | Imputed |
| **RS16940810** | 15 | 59115159 | T | -0.023 | 0.023 | 0.316 | 0.006 | 0.017 | 0.713 | Genotyped |
| **RS618684** | 15 | 59115922 | G | -0.004 | 0.007 | 0.535 | -0.007 | 0.005 | 0.163 | Imputed |
| **RS4775094** | 15 | 59115995 | G | -0.005 | 0.007 | 0.524 | -0.007 | 0.005 | 0.162 | Imputed |
| **RS57410072** | 15 | 59118085 | A | 0.017 | 0.097 | 0.862 | 0.002 | 0.086 | 0.978 | Imputed |
| **RS11629654** | 15 | 59118567 | T | 0.004 | 0.011 | 0.691 | -0.018 | 0.007 | 0.014 | Imputed |
| **RS59576117** | 15 | 59119725 | T | 0.017 | 0.097 | 0.862 | 0.002 | 0.086 | 0.978 | Imputed |
| **RS11071398** | 15 | 59120077 | G | -0.002 | 0.007 | 0.798 | -0.003 | 0.005 | 0.564 | Imputed |
| **RS4775095** | 15 | 59120626 | C | 0.007 | 0.009 | 0.41 | -0.008 | 0.006 | 0.207 | Imputed |
| **RS12901111** | 15 | 59121245 | G | 0.026 | 0.097 | 0.786 | 0.147 | 0.086 | 0.089 | Imputed |
| **RS71478693** | 15 | 59121463 | T | -0.032 | 0.068 | 0.639 | 0.04 | 0.05 | 0.424 | Imputed |
| **RS28549775** | 15 | 59121557 | C | 0.007 | 0.009 | 0.41 | -0.008 | 0.006 | 0.23 | Imputed |
| **RS12905507** | 15 | 59121613 | G | 0.006 | 0.01 | 0.581 | -0.006 | 0.007 | 0.395 | Imputed |
| **RS8029301** | 15 | 59121836 | T | -0.012 | 0.009 | 0.186 | 0.002 | 0.007 | 0.737 | Imputed |
| **RS12595057** | 15 | 59122826 | G | -0.009 | 0.01 | 0.332 | 0.001 | 0.007 | 0.837 | Imputed |
| **RS34849581** | 15 | 59123238 | A | -0.01 | 0.01 | 0.313 | 0.001 | 0.007 | 0.934 | Imputed |
| **RS449243** | 15 | 59123240 | A | -0.013 | 0.01 | 0.193 | 0 | 0.007 | 0.984 | Imputed |
| **RS12594276** | 15 | 59125005 | G | 0.007 | 0.009 | 0.41 | -0.006 | 0.006 | 0.327 | Imputed |
| **RS57285066** | 15 | 59126094 | G | 0.017 | 0.097 | 0.862 | 0.002 | 0.086 | 0.978 | Imputed |
| **RS57783587** | 15 | 59126202 | T | 0.017 | 0.097 | 0.862 | 0.002 | 0.086 | 0.978 | Imputed |
| **RS624536** | 15 | 59126498 | C | 0.008 | 0.009 | 0.352 | -0.008 | 0.006 | 0.231 | Imputed |
| **RS12914164** | 15 | 59126897 | G | 0.008 | 0.009 | 0.352 | -0.008 | 0.006 | 0.231 | Imputed |
| **RS62002455** | 15 | 59127565 | T | -0.009 | 0.01 | 0.354 | 0.004 | 0.007 | 0.56 | Imputed |
| **RS62002456** | 15 | 59127844 | G | -0.009 | 0.01 | 0.354 | 0.004 | 0.007 | 0.56 | Imputed |
| **RS12437928** | 15 | 59128019 | C | 0.008 | 0.009 | 0.352 | -0.008 | 0.006 | 0.231 | Imputed |
| **RS1551445** | 15 | 59128558 | G | -0.008 | 0.01 | 0.438 | 0.004 | 0.007 | 0.601 | Imputed |
| **RS4566091** | 15 | 59128615 | T | 0.017 | 0.097 | 0.862 | 0.002 | 0.086 | 0.978 | Imputed |
| **RS7174187** | 15 | 59129737 | T | 0.004 | 0.011 | 0.705 | -0.017 | 0.007 | 0.022 | Imputed |
| **RS11631799** | 15 | 59130927 | G | 0.004 | 0.011 | 0.705 | -0.017 | 0.007 | 0.022 | Imputed |
| **RS2054097** | 15 | 59131331 | A | -0.032 | 0.033 | 0.323 | -0.019 | 0.022 | 0.392 | Imputed |
| **RS59518143** | 15 | 59131520 | G | -0.011 | 0.01 | 0.258 | 0.007 | 0.007 | 0.335 | Imputed |
| **RS535679** | 15 | 59135495 | T | -0.005 | 0.015 | 0.74 | 0.003 | 0.012 | 0.796 | Imputed |
| **RS7178955** | 15 | 59135760 | G | -0.007 | 0.008 | 0.388 | 0.004 | 0.006 | 0.477 | Imputed |
| **RS7178522** | 15 | 59135784 | A | -0.007 | 0.008 | 0.388 | 0.004 | 0.006 | 0.477 | Imputed |
| **RS8039141** | 15 | 59135910 | G | 0.001 | 0.015 | 0.972 | 0.007 | 0.012 | 0.548 | Imputed |
| **RS813220** | 15 | 59136688 | G | -0.01 | 0.009 | 0.253 | 0.006 | 0.006 | 0.389 | Imputed |
| **RS10851640** | 15 | 59137870 | T | -0.007 | 0.008 | 0.358 | 0.003 | 0.006 | 0.56 | Imputed |
| **RS637918** | 15 | 59138270 | A | -0.01 | 0.009 | 0.253 | 0.006 | 0.006 | 0.389 | Imputed |
| **RS6494041** | 15 | 59139106 | G | -0.004 | 0.015 | 0.77 | 0.004 | 0.012 | 0.735 | Imputed |
| **RS11071399** | 15 | 59139255 | A | -0.007 | 0.008 | 0.378 | 0.004 | 0.006 | 0.477 | Imputed |
| **RS4775096** | 15 | 59140329 | C | -0.007 | 0.008 | 0.347 | 0.003 | 0.006 | 0.559 | Genotyped |
| **RS4775097** | 15 | 59140801 | C | -0.007 | 0.008 | 0.358 | 0.003 | 0.006 | 0.56 | Imputed |
| **RS4774312** | 15 | 59140924 | A | -0.008 | 0.008 | 0.343 | 0.003 | 0.006 | 0.57 | Imputed |
| **RS813219** | 15 | 59141699 | A | -0.01 | 0.009 | 0.281 | 0.006 | 0.006 | 0.325 | Imputed |
| **RS793571** | 15 | 59141706 | G | -0.008 | 0.008 | 0.341 | 0.002 | 0.006 | 0.714 | Imputed |
| **RS6494042** | 15 | 59141986 | A | 0.017 | 0.097 | 0.862 | 0.002 | 0.086 | 0.978 | Imputed |
| **RS670920** | 15 | 59142094 | G | -0.008 | 0.01 | 0.406 | 0.005 | 0.007 | 0.439 | Imputed |
| **RS72745044** | 15 | 59142170 | G | 0.019 | 0.031 | 0.53 | 0.017 | 0.021 | 0.417 | Imputed |
| **RS35536174** | 15 | 59142215 | T | -0.007 | 0.015 | 0.645 | 0.004 | 0.012 | 0.728 | Imputed |
| **RS16940850** | 15 | 59144199 | A | -0.007 | 0.008 | 0.388 | 0.004 | 0.006 | 0.477 | Imputed |
| **RS640045** | 15 | 59144461 | A | 0.002 | 0.014 | 0.907 | 0.009 | 0.012 | 0.456 | Imputed |
| **RS35059967** | 15 | 59146247 | C | 0.001 | 0.016 | 0.929 | 0.015 | 0.013 | 0.231 | Imputed |
| **RS7164683** | 15 | 59146372 | A | -0.007 | 0.008 | 0.388 | 0.004 | 0.006 | 0.485 | Imputed |
| **RS62004762** | 15 | 59146382 | A | 0 | 0.016 | 0.986 | 0.012 | 0.013 | 0.348 | Imputed |
| **RS16940856** | 15 | 59147548 | A | 0.001 | 0.016 | 0.929 | 0.015 | 0.013 | 0.231 | Imputed |
| **RS431414** | 15 | 59147800 | T | -0.011 | 0.009 | 0.213 | 0.006 | 0.006 | 0.325 | Imputed |
| **RS1054991** | 15 | 59149400 | G | -0.008 | 0.008 | 0.335 | 0.002 | 0.006 | 0.689 | Imputed |
| **RS76751232** | 15 | 59149756 | G | 0 | 0.014 | 0.982 | -0.025 | 0.01 | 0.011 | Imputed |
| **RS677933** | 15 | 59151916 | G | -0.01 | 0.009 | 0.26 | 0.006 | 0.006 | 0.334 | Imputed |
| **RS472200** | 15 | 59152402 | G | -0.007 | 0.015 | 0.653 | 0.004 | 0.012 | 0.719 | Imputed |
| **RS661367** | 15 | 59153303 | G | -0.007 | 0.015 | 0.653 | 0.004 | 0.012 | 0.719 | Imputed |
| **RS1053420** | 15 | 59153808 | A | -0.009 | 0.008 | 0.234 | 0.001 | 0.006 | 0.869 | Imputed |
